# The benefits of the Mediterranean diet in first episode psychosis patients taking antipsychotics

**DOI:** 10.1016/j.toxrep.2022.01.003

**Published:** 2022-01-10

**Authors:** Emilia Vassilopoulou, Dimitris Efthymiou, Vasilis Tsironis, Panayiotis Athanassis, Stelios Chatzioannidis, Theano Kesoglou, Andrey V. Severin, Vasilis P. Bozikas

**Affiliations:** a Department of Nutrition and Dietetics, International Hellenic University, Thessaloniki, Greece; b 2nd Department of Psychiatry, Division of Neuroscience, Medical School, Aristotle University of Thessaloniki, Thessaloniki, Greece; c Department of Analytical and Forensic Medical Toxicology, Sechenov University, 119048, Moscow, Russian Federation; d Medical School, University of Crete, Heraklion, Greece

**Keywords:** AP, antipsychotic, CRP, C-reactive protein, CVD, cardiovascular disease, DM, diabetes mellitus, FEP, First Episode Psychosis, HC, Healthy Control, HDL-C, HDL cholesterol, hsCRP, high-sensitivity CRP, LDL-C, LDL cholesterol, MedDiet, Mediterranean Diet, TG, triglycerides, First episode psychosis, Anti-psychotics, Mediterranean diet, Fermented food, Inflammation, Metabolic disturbances

## Abstract

**Background:**

The side effects of antipsychotics (APs), related to weight gain and metabolic disturbances, can contribute to the health burden of psychotic people.

**Objective:**

To explore a) the level of adherence to the Mediterranean Diet (MedDiet) and consumption of fermented foods by first episode of psychosis (FEPs) patients taking APs, in comparison to matched -for age and BMI- healthy controls (HCs), and b) the effect of this dietary pattern on the biochemical and metabolic profile of FEPs.

**Method:**

The study population consisted of 33 FEPs treated with APs for less than 5 years, with no history of other chronic diseases, and an equal number of HCs. The FEPs were classified into two subgroups, according to their AP medication, depending on the documented risk of weight gain. A validated questionnaire for the adherence to Mediterranean diet and a food frequency questionnaire for selected fermented foods were completed by FEPs and HC. Anthropometric data and blood measurements were recorded for all participants.

**Results and conclusions:**

The FEPs showed a relevant lower overall adherence to the MedDiet, but no differences in consumption of fermented foods. Type of antipsychotic therapy uncovered differences in platelet count, vitamin B12, HDL and glucose (p < 0.05) between the subgroups of FEPs and HCs, although no values were abnormal. The MedDiet score was found to act as a prognostic factor for abnormal glucose levels in FEPs treated with APs associated with weight gain (*p* = 0.04). These results need to be confirmed by observations after long term adherence to MedDiet.

## Graphical abstract

**Figure:**
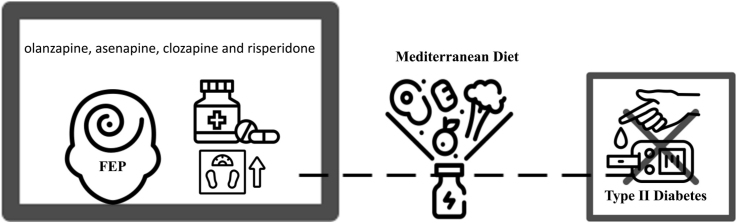

## 1 Introduction

Pharmacological intervention is the main treatment option for young people presenting psychotic manifestations for the first time [1]. Well documented evidence shows second-generation antipsychotics (APs) to be comparable in efficacy to, if not more effective than, first-generation APs, but with better tolerability and less adverse effects [2]. More than half of the patients in one study showed symptomatic remission and one quarter (26.4 %) functional remission, while one-fifth (19.2 %) recovered completely after receiving second generation AP treatment [3].

The side effects of APs, however, and in particular, weight gain, obesity, and metabolic disturbances, can constitute significant contributors to the health burden of people with psychosis. Subcutaneous and intra-abdominal fat may increase significantly after the initiation of AP treatment [4], and increases in the levels of circulating lipids and non-fasting glucose have been observed in all patients, but especially in women [5]. Consequently, the risk of cardiovascular disease (CVD) and type 2 diabetes mellitus (DM) is increased by two to three times in patients with schizophrenia taking APs, with a reduction in life expectancy by one fifth, compared with the general population [6].

All APs have been found to be correlated with the induction of metabolic complications and weight gain [7] but aripiprazole, amisulpride, quetiapine, paliperidone and ziprasidone appear to induce less intense effects than olanzapine, asenapine, clozapine and risperidone [8,9].

The etiology of the metabolic disturbances in schizophrenia is multifactorial and may be associated not only with AP treatment, but also with high levels of stress, a poor diet, and reduced physical activity [10].

First-episode psychosis (FEPs) patients should be distinguished from patients with chronic psychiatric disease [11] as the former appear to be more vulnerable to weight gain and drug related metabolic disturbances [8]. In addition, after the initiation of AP treatment, the dietary habits of FEPs are characterized by a high intake of saturated fat and refined carbohydrates, and low consumption of fiber and fruit [12]. Low adherence to healthy dietary patterns, such as the Mediterranean diet (MedDiet) has been observed in a high proportion of FEPs (60 %) [13].

A healthy lifestyle, including a balanced diet, such as MedDiet [14], is recommended for patients with mental disorders, and regular exercise, and smoking cessation are also documented to reduce their metabolic and cardiovascular risk [15]. In addition, the prophylactic effect of gut microbiome enrichment with probiotics via fermented foods has been demonstrated [16].

Nutritional psychiatry has generated reliable evidence that through the gut-brain axis interactions, a diet rich in antioxidants and low processed natural foods, such as fermented foods, is a modifiable factor for the promotion of the health of mind and body [17], but clinicians often refer to dietary intervention for patients with psychosis as resembling a hurdle race [16].

MedDiet has consistently been associated with reduction in the levels of various coagulation and inflammation markers related to CVD, including C-reactive protein (CRP) and platelets [18], and a healthy anti-inflammatory diet might therefore be protective against the metabolic complications of psychosis and AP treatment. This observational study aimed to explore: a) the level of adherence to the MedDiet and consumption of selected fermented foods by FEPs with psychosis, in comparison to matched healthy control subjects (HCs), and b) the associations of MedDiet, fermented foods and APs on the anthropometric and biochemical/metabolic profile of FEPs, in comparison to HCs.

## 2 Methodology

### 2.1 Study population

FEPs diagnosed with psychosis at the 2nd Psychiatric University Clinic of the Aristotle University of Thessaloniki and its Early Intervention Outpatients services in Thessaloniki, Greece, and receiving APs for less than five years, were recruited into the study.

Inclusion criteria were: 1) diagnosis of a psychotic episode according to the DSM-5 criteria, 2) age between 18–60 years, and 3) AP medication of less than 5 years’ duration. Exclusion criteria: were 1) a clinical history of substance misuse (dual diagnosis patients), 2) AP medication for more than 5 years, 3) pregnancy, 4) mental retardation, 5) chronic medical/metabolic conditions, such as CVD and DM, 6) medication with a known effect on blood glucose and lipids.

The HC study sample consisted of individuals without a diagnosis of mental illness or chronic metabolic disease, matched for sex, age, and body mass index (BMI) with the FEP group.

Before inclusion in the study, all the participants were informed in detail about the study protocol and provided their written informed consent. The study was approved by the Research Ethics Committee of the Aristotle University of Thessaloniki (code number 3.303) and complied with the International Code of Medical Ethics of the World Medical Association and the Helsinki Declaration.

### 2.2 Clinical assessment

Classification of the mental disorder of the FEPs was made according to the ICD-10 classification by the medical team of the Early Intervention Outpatient Clinic and cross-checked by one of the psychiatrists in the study team. The AP prescribed to each of the FEPs in the study was recorded in detail and subdivided into two categories: AP1, related to a < 20 % risk of weight gain (WG) of ≥7% from the baseline weight, namely aripiprazole, amisulpride quetiapine XR, paliperidone, and ziprasidone, and AP2, related to a > 20 % risk of WG ≥ 7%, namely olanzapine, asenapine, clozapine and risperidone [[7], [8], [9]]. The FEPs were categorized into two subgroups, according to the AP prescribed: AP1 FEP and AP2 FEP, respectively. A detailed medical and family history was recorded, with a focus on chronic metabolic diseases, such as CVD and type II DM, and a family history of psychosis in first degree relatives.

### 2.3 Blood pressure determination

Arterial blood pressure (BP) was recorded to the nearest 2 mmHg, using a mercury sphygmomanometer with the arm supported at heart level, after the subject had been sitting quietly for 10 min. One trained member of the research team took three separate readings at 1-min intervals in the morning of the visit to the hospital. The average of the last two readings was used for analysis.

### 2.4 Biochemical/hematological assessment

Venous blood samples were collected from all participants, as part of their routine follow up procedure, after overnight fasting, and analyzed on the hospital premises, using automatic biochemical analyzers, under standard conditions, then stored at −80 °C until further analysis. The concentrations of plasma glucose, serum total cholesterol, triglycerides (TG), HDL cholesterol (HDL-C), LDL cholesterol (LDL-C) and high-sensitivity CRP (hsCRP) were measured by automatic analyzer (Toshiba TBA 120FR; Toshiba Medical Systems Co., Ltd., Tokyo, Japan). Glycated hemoglobin (HbA1c) was measured by automatic analyzer (HLC-723 G7 HPLC systems; Tosoh Corporation, Tokyo, Japan).

### 2.5 Anthropometric assessment

Anthropometric measurements were made on all participants in the morning, after fasting for at least 8 h, by one trained investigator. Height was measured to the nearest 0.1 cm using a commercial stadiometer (Leicester Height Measure, Invicta Plastics Ltd, Oadby, UK) with the participants barefoot, their shoulders in a relaxed position, their arms hanging freely and their heads in the Frankfort horizontal plane. The waist circumference (WC) was measured with a SECA flexible, inextensible measuring tape with an accuracy of 1 mm, on a horizontal plane, after exhalation, at a point equidistant from the lowest floating rib and the upper border of the iliac crest. The participants were weighed barefoot and in light clothing to the nearest 0.1 kg by using a TANITA RD-545 [19]. TANITA RD-545 is equipped with a Biometric Impedance Analysis system (BIA), providing information on fat and muscle mass for the overall body composition and separately for arms, legs, and truck, BMI [weight (kg) by height squared (m2)], body water, basal metabolic rate (BMR), metabolic age, visceral fat, muscle mass, muscle quality score, percentage of body fat (% BF) and physical rating.

### 2.6 Dietary assessment

Adherence to the MedDiet was assessed using the validated Mediterranean Diet Score questionnaire [20]. This questionnaire lists 11 main components of the MedDiet, for which the participants were requested to report the frequency of consumption. A composite MedDiet score was calculated as follows: for the components commonly consumed in the MedDiet (non-refined cereals, potatoes, fruits and fruit juice, vegetables, and salad, legumes, and fish), a score of 0 (lowest adherence) to 5 (highest adherence) was assigned, for reported consumption of 0, 1–4, 5–8, 9–12, 13–18 and >18 servings/month, respectively. For the components which are less frequently consumed in the MedDiet (red meat and meat products, poultry, and whole-fat dairy products), a score of 0–5 was assigned for reported consumption, using a reverse scale. A score of 0–5 was assigned for using olive oil (in meal preparation and cooking) ‘never’, ‘rarely’, ‘>1 time/week’, ‘1–3 times/week’, ‘3–5 times/week’ and ‘daily’, respectively.

For alcohol (all alcoholic beverages), a score of 5 was assigned for ‘no consumption’ or ‘consumption of <300 mL/day’, a score of 0 for ‘consumption of >700 mL/day’, and scores of 4 to 1 for the consumption of ‘300–400 mL’, ‘400–500 mL’, ‘500–600 mL’ and ‘600–700 mL/day’, respectively. The resulting total score ranged from 0–55, with calculated tertiles indicating low (0–13), moderate (14–27), good (28–41) and high (42–55) adherence to the MedDiet [18,20].

### 2.7 The Metabolic syndrome

The metabolic syndrome (MetS) was defined according to the International Diabetes Federation (IDF) criteria [21].

### 2.8 Statistical analysis

Quantitative variables were presented using mean values ± standard deviation (± SD) or median values and interquartile range (IQR), according to the normality of data. Normality of data was assessed with Kolmogorov-Smirnov test. Independent student’s *t*-test was used for the comparison of mean values when the distribution was normal and Mann-Whitney test for the comparison of median values when the distribution was not normal. To assess the difference of anthropometric characteristics and biochemical indicators according to the subgroups of participants (AP1, AP2 or HC) univariate analyses of variance (one way ANOVA) or Kruskall Wallis criterion were implemented. Multiple comparisons were conducted with Bonferroni tests (if the initial test was ANOVA) or Dunn pairwise tests adjusted using Bonferroni correction (if the initial test was Kruskall-Wallis). Regression analysis was performed for each AP group with the dependent variable one of the biochemical variables (platelets, B12, HDL, glucose) each time and the MedDiet score the independent variable. Statistical analysis was conducted using IBM SPSS Statistics version 21.0 for Windows (IBM, Armonk, NY, US). Statistical significance was set at *p*-value < 0.05.

## 3 Results

A total of 33 subjects diagnosed with a psychotic episode according to DSM-5 criteria within the last 5 years (mean years since diagnosis 3.2 ± 1.8), 22 men and 11 women (the FEP group), were recruited from October 2019 to March 2020, along with 33 HC subjects, matched for age, sex, and BMI. The comparative demographic characteristics and anthropometric measurements of the participants are presented in Table 1. The age and anthropometric measurements were similar between the two groups, in both sexes. All the subjects in both groups were borderline for overweight (BMI > 24.9), with a physique rating higher than 3, indicating increased fat mass. In both FEP and HC groups the estimated metabolic age was higher than the actual age, showing that the subjects were physically inactive. Only two FEP females and one HC female were diagnosed with MetS, so no further analysis was performed regarding MetS in our sample.

**Table 1:** Demographic characteristics of the study population of patients with a first episode of psychosis (FEP) (n = 33) and healthy control subjects (HC) (n = 33).

	MALES			FEMALES		
	(Mean ± SD)		P- value	(Mean ± SD)		P- value
	FEP	HC		FEP	HC	
Age (yrs)^ｷ^	29.50 (14.00)	32.00 (15.00)	0.527^2^	35.00 (17.00)	35.00 (17.00)	0.878^2^
Weight (kg)	83.63 ± 16.46	85.84 ± 14.18	0.640^1^	70.98 ± 17.61	65.08 ± 12.12	0.357^1^
Height (m)	1.80 ± .06	1.83±.12	0.327^1^	1.66±.07	1.65±.06	0.614^1^
BMI (kg/m^2^)	25.72 ± 5.44	25.76 ± 3.69	0.978^1^	25.10 ± 6.32	24.02 ± 4.33	0.637^1^
WC (cm)	93.6 ± 7.2	92.4 ± 6.9	0.654^1^	86.4 ± 8.9	85.1 ± 8.4	0.556^1^
% BF	24.22 ± 8.32	22.95 ± 6.79	0.586^1^	31.62 ± 8.61	29.32 ± 8.40	0.525^1^
Muscle mass (kg)	59.16 ± 6.56	62.20 ± 7.32	0.160^1^	44.80 ± 6.17	43.09 ± 5.10	0.475^1^
Muscle Quality score	48.41 ± 9.73	52.95 ± 10.52	0.149^1^	49.64 ± 6.76	52.67 ± 10.17	0.414^1^
Physique rating^ｷ^	2.00 (2.00)	2.00 (3.00)	0.395^2^	3.00 (3.00)	4.5 (4.00)	0.444^2^
Bone mass (kg)	3.11 ± .32	3.28±.34	0.096^1^	2.39±.33	2.31±.27	0.559^1^
Visceral fat rating^ｷ^	7.00 (7.63)	8.50 (6.75)	0.874^2^	4.5 (6.00)	3.25 (5.75)	0.975^2^
BMR (kcal)	1855.05 ± 229.66	1954.57 ± 229.14	0.163^1^	1447.18 ± 203.05	1380.00 ± 155.11	0.380^1^
Metabolic age (yrs)	37.32 ± 14.67	39.05 ± 15.04	0.705^1^	37.09 ± 18.19	36.92 ± 16.71	0.981^1^
Body water (kg)	56.46 ± 7.06	56.52 ± 4.71	0.972^1^	50.87 ± 7.05	51.75 ± 5.35	0.739^1^
Systolic BP (mmHg ) ^ｷ^	120.00 (8.00)	120.00 (5.00)	0.781^2^	115.00 (11.00)	120.00 (15.00)	0.167^2^
Diastolic BP (mmHg)^ｷ^	75.00 (8.00)	75.00 (8.00)	0.969^2^	75.00 (3.00)	75.00 (14.00)	0.891^2^

^1^Independent samples T-test, ^2^Mann-Whitney U, ^ｷ^Median and IQR.

BMI: body mass index, WC: waist circumference, BF: body fat, BMR: basic metabolic rate, BP: arterial blood pressure.

The scores on the MedDiet questionnaire are shown in Table 2. The HC group presented a significantly higher overall median score, of a level indicating good adherence to the MedDiet, while the FEP group showed a moderate median level of adherence (29.00 (IQR = 8.00) for HC vs 27.00 (IQR = 4.00) for FEP; *p* = 0.002). The HC group consumed significantly more whole wheat products (median=1.00 for HC vs median=0.00 for FEP) and legumes (median = 2 for both groups with values for HC found to vary from 0 to 3 and for FEP from 1 to 2). Although not to a statistically significant degree, HC consumed less red meat and drank more alcohol in comparison with FEP. Consumption of fermented foods was low and similar in the two groups (*p* > 0.05) (Table 2).

**Table 2:** Adherence to the Mediterranean diet, according to the MedDiet score of the population of patients with a first episode of psychosis (FEP) (n = 33) and healthy control subjects (HC) (n = 33).

	FEP (Median; IQR)	HC (Median; IQR)	P-value
**Whole wheat/Non fermented starch products**	0.00 (1.00)	1.00 (1.00)	**0.002**
Potatoes	2.00 (2.00)	2.00 (1.00)	0.332
Fruit	2.00 (2.00)	2.00 (1.00)	0.487
Vegetables	1.00 (1.00)	1.00 (1.00)	0.779
**Legumes**	**2.00 (1.00)**	**2.00 (1.00)**	**0.040**
Fish	1.00 (1.00)	1.00 (1.00)	0.110
Red meat	5.00 (1.00)	4.00 (1.00)	0.554
Chicken	5.00 (.00)	5.00 (.00)	0.619
Dairy	5.00 (1.00)	5.00 (1.00)	0.742
Olive oil	5.00 (.00)	5.00 (.00)	0.648
Alcohol	.00 (.00)	.00 (2.00)	0.071
**MedDiet total score**	**27.00 (4.00)**	**29.00 (6.00)**	**0.002**
**Selected Fermented products**			
Yogurt with probiotics	0.00	0.00	–
Traditional Greek yogurt	0.00	0.00	–
Cheese	0.00 (.00)	0.00 (.00)	0.517
Olives	2.00 (2.00)	3.00 (3.00)	0.487
Pickled vegetables	1.00 (2.00)	1.00 (1.00)	0.388

Note: Statistically significant differences marked in bold.

Subsequently, the FEPs were subdivided into two subgroups, AP1, with 15 patients and AP2, with 18 patients, according to the specific AP they were taking (see above). No statistically significant difference was demonstrated in the anthropometric measurements between the FEP subgroups and the HC group, even after adjustment for the MedDiet score and consumption of fermented foods (results not shown).

As shown in Table 3, significant differences in mean blood levels of various parameters were demonstrated between the HC group and the FEP group according to their AP, despite their similar body phenotype. Among these parameters only glucose was marginally abnormal in AP2 patients (median 100.69 (22.00) mg/dL). Specifically, the platelet count (*p* = 0.022), and the serum levels of B12 (*p* = 0.011), urea (*p* ≤ 0.001), creatinine (*p* = 0.023), SGOT (*p* = 0.002), HDL (*p* = 0.002) and glucose (*p* = 0.017) showed significant differences between AP1 FEP and HC. Between AP2 FEP and HC, significant differences were observed in urea (*p* ≤ 0.001) and HDL (*p* = 0.002), and between the AP1 and AP2 subgroups in glucose (*p* = 0.033).

**Table 3:** Biochemical indices in the population of patients with a first episode of psychosis (FEP) (n = 33), taking antipsychotics (AP1 or AP2)*, and healthy control subjects (HC) (n = 33).

	AP1 n = 15 (Mean ± SD)	AP2 n=18 (Mean ± SD)	HC n = 33 (Mean ± SD)	P-value
Ht (% red cells/blood)	41.94 ± 3.06	42.78 ± 4.30	41.54 ± 4.06	0.577^1^
RBC (cells* 10^3^ mcL)	4.73 ± .53	5.09 ± 0.51	4.86±.48	0.117^1^
WBC (cells*10^9^/L) ^ｷ^	7.04 (2.31)	6.93 (3.65)	6.43 (1.42)	0.135^2^
Pts (cells*10^9^/L)	**263.54 ± 56.68**	246.71 ± 56.19	**220.75 ± 36.19**	**0.017^1^**
Iron (μg/dL)	66.00 ± 23.80	91.75 ± 43.38	75.05 ± 32.09	0.349^1^
Ferritin (ng/L) ^ｷ^	62.00 (67.00)	128.00 (121.00)	59.00 (62.00)	0.121^2^
**B12 (ng/L)**	**232.40 ± 30.24**	316.00 ± 169.93	**415.33 ± 70.42**	**0.010^1^**
Urea (mg/dL)	24.30 ± 5.72	25.33 ± 6.41	37.71 ± 6.69	≤.001^1^
Uric acid (mg/dL)	5.80 ± 1.57	5.41 ± 1.68	5.45 ± 1.63	0.936^1^
Crt (mg/dL)	.78 ± .09	.81±.13	.94±.19	0.009^1^
SGOT (U/L) ^ｷ^	14.00 (6.00)	16.50 (10.00)	19.00 (5.00)	0.006^2^
SGPT (U/L)	16.18 ± 9.41	19.13 ± 12.62	20.23 ± 6.85	0.463^1^
LDH (U/L)	123.22 ± 50.16	148.88 ± 33.90	145.75 ± 11.27	0.391^1^
γ-GT (U/L) ^ｷ^	12.50 (16.00)	13.00 (16.00)	15.00 (4.00)	0.778^2^
CRP (mg/L) ^ｷ^	.13 (.20)	.20 (.36)	.08 (.01)	0.136^2^
TG (mg/dL) ^ｷ^	93.00 (87.00)	110.00 (77.00)	86.00 (49.00)	0.819^2^
T-Chol (mg/dL)	176.73 ± 23.96	184.25 ± 40.81	179.42 ± 18.52	0.728^1^
**HDL (mg/dL)**	**45.93 ± 7.89**	**46.56 ± 8.38**	**57.63 ± 11.73**	**≤.001^1^**
LDL (mg/dL)	118.93 ± 29.63	120.25 ± 37.72	106.81 ± 27.16	0.260^1^
**Gluc** **(mg/dL) ^ｷ^**	**83.00 (14.00)**	**100.69 (22.00)**	**94.00 (13.00)**	**0.041^2^**

^1^ANOVA, ^2^ Kruskal-Wallis, ^ｷ^Medians and IQRs are presented.

*AP1: Antipsychotics with lower risk of weight gain; AP2: Antipsychotics with higher risk of weight gain (see text).

Ht: Hematocrit; RBC:Red Blood Cells; WBC:White Blood Cells; Pts: Platelets; Crt: Creatinine; SGOT: Serum Glutamic-Oxaloacetic Transaminase; SGPT: Serum Glutamic Pyruvic Transaminase; LDH: lactate dehydrogenase; γ-GT: gamma-glutamyltransferase; CRP:C-Reactive Protein; TG:Triglycerides ; T-Chol: Total cholesterol; HDL: High-density lipoprotein cholesterol; LDL: Low-density lipoprotein cholesterol; Gluc: Glucose.

Note: Statistically significant differences marked in bold.

In linear regression analysis, the MedDiet score was associated only with the glucose level in AP2; for every unit of increase in MedDiet score the level of glucose dropped by approximately 2.43 units (b = -2.43, *p* = 0.041) (Table 4).

**Table 4:** Linear regression analysis with biochemical indicators as the dependent variable in first episode psychotic patients (FEPs): A) AP1 FEPs: B) AP2 FEPs.

A)												
AP1	Pts (n = 13)			B12 (n = 5)			HDL (n = 15)			Gluc (n = 14)		
	***β+***	***SE++***	***p***	***β+***	***SE++***	***p***	***β+***	***SE++***	***p***	***β+***	***SE++***	***p***
Med Diet Score	1.04	6.41	0.874	1.00	7.23	0.899	−0.72	0.80	0.386	1.63	1.57	0.320
*R^2^*	0.002			0.006			0.058			0.082		
*F*	0.026			0.019			0.803			1.075		
*p-value*	0.874			0.899			0.386			0.320		

B)												
AP2	Pts (n = 17)			B12 (n = 7)			HDL (n = 16)			Gluc (n = 14)		
	***β+***	***SE++***	***p***	***β+***	***SE++***	***p***	***β+***	***SE++***	***p***	***β+***	***SE++***	***p***
Med Diet Score	−.96	2.23	0.672	−5.89	14.53	0.702	0.06	0.34	0.863	−2.43	1.08	**0.041**
*R^2^*	0.012			0.032			0.002			0.266		
*F*	0.187			0.164			0.031			5.071		
*p-value*	0.672			0.702			0.863			**0.041**		

+ unstandardized beta coefficient, ++ standard error of the unstandardized beta coefficient.

In bold if statistically significant at p ≤ 0.05 or p ≤ 0.001.

AP1: Antipsychotics with lower risk of weight gain; AP2: Antipsychotics with higher risk of weight gain (see text); HC: Healthy control subjects.

Pts: Platelets; HDL: High-density lipoprotein; Gluc: Glucose.

## 4 Discussion

This study aimed to identify the possible association of disturbances in the metabolic profile and inflammatory indices of FEPs according to their AP treatment, and to explore the potential protective role of MedDiet and the consumption of selected fermented foods. Differences were observed in the mean levels of several inflammatory and metabolic indices between the two FEP subgroups groups taking different APs, and the HCs, although the values were not abnormal. Despite the other sub-clinical differences, only abnormal glucose levels in patients treated with anti-psychotics related to increased weight gain, appeared to be affected with the adherence to MedDiet.

A decade ago, Thornicroft labeled the unilateral therapeutic model for mental disorders factors, which he believed were not sufficiently considered, and which reduce the life expectancy of patients treated for psychosis by approximately two decades [22]. Gut microbiome, dietary modification, and association with immune, metabolic and psychopathological functioning in psychosis is recognized, but not well characterized [23]. Among others, the MedDiet pattern, mostly due to the high fiber content, is proposed for halting the metabolic and immune related complications of schizophrenia and APs use [23,24]. The FEPs in this study, although living in the Mediterranean basin, reported only moderate adherence to the MedDiet compared with their matched HCs. Notwithstanding, lower scores on the MedDiet scale were due mainly to the lower consumption of whole wheat products and pulses, both well documented for promoting beneficial gut microbiota composition and protecting from inflammation [25]. The olive oil effect could not be further evaluated in the under-study population, as it was similar in comparison with the HC group. Recently though its overall antioxidant effect was disputed, as Kouka et al. proposed that it can induce oxidative stress in some tissues, namely spleen, pancreas, liver and heart [26]. Presumably, the continuous inflammation in the gut-brain axis in FEPs is linked with the Western-type diet, with less pulses and fish, more meat and meat products, highly refined starchy products without their intact fibers, convenience foods and sweetened beverages - all high in calories, sugars, fats- including olive oil, trans fats, and saturated fats- salt and other food additives [27].

The unhealthy dietary habits of FEPs can be partly justified by their treatment with APs, which, on interaction with serotonergic, histaminergic and dopaminergic neurotransmitter systems, provoke changes in appetite and food intake [28,29]. Saugo et al. suggested early dietary and lifestyle interventions in FEPs, as Aps side effects reveal withing the first months of the initiation of AP treatment [13].

In a “frugal” model of the psychosis etiology, the condition has been described as partly the result of a homeostatic signaling imbalance, caused by metabolic abnormalitites, increased inflammation and immune dysregulation [30]. Apart from the nature of the disease itself, the APs administered for its treatment induce various severe metabolic disturbances that, in combination with the disease, create a deleterious environment [31].

It is recognized that minor increases in inflammation constitute a precursor for several chronic metabolic-related conditions, including obesity, CVD and type 2 DM, even in otherwise healthy individuals [24].

The FEP group in this study, in comparison with the HC group, presented subclinical differences in a cluster of inflammatory and metabolic markers, sometimes associated with the AP medication. Specifically, in the FEP AP1 group, the platelet count was higher; in addition to their role in hemostasis and thrombosis, platelets are related to the production of several inflammatory markers [32]. The higher glucose levels in the FEP AP2 subgroup may be indicative of a sub-clinical oxidative stress response of the pancreatic cells, also related to the reduced adherence to the MedDiet. In a study of Hsu et al., clozapine categorized here in the AP2, was associated with glucose dysregulation in a mouse model [33]. In FEP treated with AP2, MedDiet appeared to be beneficial in regulating glucose levels [34].

The significantly lower mean levels of vitamin B12 in all FEPs compared with the HC group is a further signal of their sub-clinical disturbed metabolism. Low levels of B12 have been linked with the pathophysiology of schizophrenia, and specifically with the raised levels of homocysteine and cortisol in untreated subjects [35]. Notably, in the current study, the FEPs consumed more red meat products than the HCs, although not to a statistically significant degree, constituting their main source of B12 [36], and therefore enhancing disturbances in the metabolic processes.

In addition, both subgroups of FEPs had lower HDL levels than the HCs. Suboptimal levels of HDL, apart from increasing the risk of CVD complications [37], have been considered to play a role on brain myelination, with an increase of negative symptoms [38].

One significant limitation of this study was that, as it was cross-sectional in nature, it included only one-point measurements, which restricted our ability to determine a cause-and-effect relationship. For this reason, we cannot proceed to definite conclusions on whether the pro-inflammatory and metabolic differences were related to the disease and/or the AP therapy. Because of the small sample size, we could not formulate definite conclusions on the different effects of the two categories of AP, although some were apparent. In addition, as the FEPs had been taking an AP medication for an average of 3.3 years, no information could be extracted regarding the initial drug-naive phase.

## 5 Conclusions

Overall, the results of this study are suggestive that in well-controlled FEPs taking AP2, which are documented to be associated with higher weight gain, an increase in MedDiet adherence can benefit glucose modulation. The FEPs in this study taking APs presented low-grade systemic inflammation, presumably interdependent with metabolic-related conditions. This metabolo-immunologic profile can be compared with a “sleeping volcano preparing for eruption”. Our results are promising regarding the potential of MedDiet for balancing the metabolism disturbed by APs, and restoring anti-inflammatory and antioxidant depositories. Long-term personalized dietary intervention in the spectrum of MedDiet for FEPs treated with APs could provide more powerful conclusions.

## Declaration of fundings

No funding was received for the current study.

## Declaration of financial/other relationships

None by all authors.

## Authors’ contributions

EV and DE equally conceived and designed the analysis and collected the data; EV, DE, PA, SC, TK, VB contributed data or analysis tools; VT Performed the analysis; EV, DE Wrote the paper; All authors approved the paper.

## Declaration of Competing Interest

The authors declare that they have no known competing financial interests or personal relationships that could have appeared to influence the work reported in this paper.
